# Effects of Insecticides on the Fluidity of Mitochondrial Membranes of the Diamondback Moth, *Plutella xylostella*, Resistant and Susceptible to Avermectin

**DOI:** 10.1673/031.008.0301

**Published:** 2008-01-09

**Authors:** J. Hu, P. Liang, X. Shi, X. Gao

**Affiliations:** Department of Entomology, China Agricultural University, Beijing 100094, China

**Keywords:** insecticide resistance, fluorescence polarization, cross-resistance

## Abstract

The effects of various insecticides on the fluidity of mitochondrial membranes and cross-resistance were investigated in the diamondback moth, *Plutella xylostella* (L.) (Lepidoptera: Plutellidae) using strains that were both resistant and susceptible to avermectin. The resistant strain of *P. xylostella*, AV-R, developed 1078-fold resistance to avermetins with a high level of cross-resistance to the analogs of avermectins, ivermectin and emamectin benzoate. It had more than 1000 times greater resistance when compared with the avermectin-susceptible strain, XH-S. Mitochondrial membrane fluidity was measured by detecting fluorescence polarization using DPH (1,6-Diphenyl -1,3,5-hexatriene) as the fluorescence probe. Abamectin, emamectin benzoate, ivermectin, cypermethrin and fenvalerate decreased the fluidity of mitochondrial membranes in the XH-S strain at 25°C. However, fipronil and acephate did not change the fluidity of mitochondrial membrane when the concentration of these insecticides was 1×10^-4^ mol/L. Membrane fluidity increased as the temperature increased. The thermotropic effect on the polarization value of DPH increased as the insecticide concentration was increased. There was a significant difference of mitochondrial membrane fluidity between both XH-S and AV-R when temperature was less than 25°C and no difference was observed when the temperature was more than 25°C. The low-dose abamectin (0.11 mg/L) *in vivo* treatment caused a significant change of membrane fluidity in the XH-S strain and no change in the AV-R strain. However, a high-dose abamectin (11.86 mg/L) resulted in 100% mortality of the XH-S strain. *In vivo* treatment may cause a significant change of membrane fluidity in the AV-R strain

## Introduction

The diamondback moth, *Plutella xylostella* (L.) (Lepidoptera: Plutellidae), is the most destructive cosmopolitan pest of cruciferous crops (Talekar and Shelton 1993). It is one of the 20 most insecticides resistant insect species reported (Mota-Sanchez et al. 2002). Many pest insects possess a considerable potential for developing resistance to many different insecticides, including synthetic organic insecticides, *Bacillus thuringensis* (*B.t*.) (Tabashnik et al. 1990; Shelton et al. 1993), and abamectin (Wright et al. 1995; Iqbal et al. 1996). The avermectins are a family of macrocyclic lactones isolated as natural fermentation products from *Streptomyces avermitilis* (Kim and Goodfellow 2002; Chabala et al. 1980). Abamectin, ivermectin and emamectin benzoate all are semisynthetic avermectin analogs with unprecedented efficiency and breadth of effects against veterinary parasites and plant pests. Since the introduction of analogs in 1981 they have had a major impact on crop protection (Cambell 1989). With the wide and extensive use of avermectins for pest control, high resistance to this drug has developed in veterinary parasites and pests (Clark et al. 1995; Sayyed et al. 2004). It is critical to understand the resistance mechanism in order to manage resistance pest.

The main targets of avλermectin are glutamate-gated chloride channels (Ludmerer et al. 1999; Smith et al. 2000). These ion channels are responsible for facilitating selective uptake or release of ions from the cell. They are integral membrane proteins that allow for fast transmembrane passive diffusion flow of selected inorganic ions via a hydrophilic pore at around 10,000,000 ions/channels (Ana et al. 2001). The GABA (γ-aminobutyric acid) receptor is the main target for several kinds of pesticides, including dieldrin, fipronil and avermectins. These pesticides show inhibitory actions on ion channels and are highly hydrophobic compounds and probably partition into the hydrophobic environment of lipids and may also bind to the hydrophobic domains of membrane proteins.

Insecticides acting on ion channel have been shown to partition into membranes and cause changes in membrane fluidity (Joe and Lee 1985; Antunes-Madeira et al. 1990a; Blasiak 1992; Antunes-Madeira et al. 1994; Rosita et al. 2002; Cinzia et al. 2003). Avermectins are highly hydrophobic compounds suggesting that their action in biological membranes might be related to association with integral proteins and with phospholipids (Michelangeli et al. 1990). Extensive studies on the interaction of insecticides with membranes have been carried out for DDT (Joe and Lee 1985; Antunes-Madeira et al. 1990a, 1990b;) and parathion (Blasiak 1992; Antunes-Madeira et al. 1994). The effects of pyrethroids on membrane fluidity have been also studied (Rosita et al. 2002; Cinzia et al. 2003). Pyrethroids seem to stabilize gating particles of the sodium channel (Salgado and Narahashi 1993), resulting in slow closing of the sodium ion gates (Chinn and Narahashi 1986), and shifting the voltage dependence of the gates in the hyperpolarizing direction (Tatebayashi et al. 1994; Tabarean, and Narahashi 1998). Many studies showed that abamectin and its analogs acted on the complex of GABA-chloride ion channels (Ludmerer et al. 1999; Smith et al. 2000). However their effects on membrane fluidity are not clear. In this paper, we analyzed the effects of avermectins on the thermotropic properties and fluidity of mitochondrial membranes from susceptible and resistant strains of *P. xylostella*. These results were very useful to understand the mechanism of avermectin action and its relationship to insecticide resistance.

## Materials and Methods

### Insects

An abamectin sensitive *P. xylostella*, population (XH-S) was reared continuously in the laboratory without exposure to insecticides. Rearing conditions were 27±1°C, 70–90% relative humidity, and photoperiod of 16:8 L:D. The resistant strain (AV-R) was reared under the same conditions but the 3rd instar larvae of every generation was selected with abamectin.

### Chemicals

Abamectin (95% purity), ivermectin(98.9% purity), emamectin benzoate (97.2% purity) were obtained from Hisun Chemical Co. (www.hisunchem.com). Endosulfan (90% purity) and acephate (95% purity) were from Danyang Agrochemicals Co. (www.danyangchem.com) and donated by Beijing Jingnong Co. Cypermethrin (93.2% purity), Fenvalerate (96.1% purity), were obtained from Jiangsu Yangnong Chemical Group Co. (www.yangnong.com.cn). Coomassie brilliant blue G-250 was purchased from Sigma (www.sigmaaldrich.com). DPH (1,6-Diphenyl -1,3,5-hexatriene, 99% purity) was purchased from Fluca (www.sigmaaldrich.com).

### Leaf dip bioassays

Toxicity of insecticides was measured using a leaf-dip bioassay method similar to that described by Shelton *et al*. (1993). Cabbage leaves were first washed thoroughly with distilled water containing 1 g/L Triton X-100 and dried. Leaf discs of diameter 8 cm were cut and dipped in dispersions of different concentrations of the insecticide (0.00625–64mg/L). Each disc was dipped for 10 s and allowed to air-dry for a period of 1 h. The discs were then placed individually into plastic Petri dishes (diameter 9 cm). Ten to fifteen third-instar *P. xylostella* larvae were placed in each dish and three replications were prepared, resulting in a total of 30–45 larvae per treatment. Seven to twelve treatments and one control with distilled water containing 1 g/L Triton X-100 were tested in each bioassay. Larvae were allowed to feed for 48 h at 27°C at about 70–80% RH before mortality was recorded. Control mortality for all leaf dip bioassays was <5%.

### Preparation of mitochondria membrane

Mitochondrial membranes of *P. xylostella* larvae were isolated by differential centrifugation according to Voss et al (1961), using a sucrose extraction medium consisting of 10 mM Tris-HCl, 0.1 mM EDTA, 250 mM sucrose, NaCl 0.8g %, BSA 0.5g %, pH7.4. The *P. xylostella* larvae were frozen at -80°C in an ultra low temperature freezer and a group of fourth-instar larvae (body weight 9–11g) was homogenized in 2 ml of ice-chilled sucrose extracting solution with a hand-operated tissue grinder (Teflon pestle). The homogenate was centrifuged 3,000g for 10min and supernatant at 10,000g for somin at 4°C (using an Eppendorf centrifuge, 5417R, www.eppendorf.com). The sediment was suspended in 2ml ice-chilled sucrose extracting solution and then centrifuged 10,000g for 20 min and finally suspended in 2 ml ice-chilled sucrose extracting solution and kept in a refrigerator (-20°C). Mitochondrial protein was determined by the method of Bradford using bovine albumin as a standard (Bradford 1976). The protein concentration of mitochondria in the final sucrose extracting solution was about 0.8 mg protein/ml.

### Preparation of DPH and pesticide incorporation into *P. xylostella*, mitochondrial membrane

The applied concentration of DPH was based on previous reports (Ohyashiki et al. 1992; Tanii et al. 1995), and the DPH stock solution (2 mM) was prepared in tetrahydrofuran and was diluted 1000-fold by injection into a vigorously stirred solution of PBS (10 mM buffer, pH 7.4, 0.14 mol/L NaCl), and stirred for 1 h before use. Briefly, the diluted mitochondrial membranes were mixed with the fluorescent probe DPH (0.3 mg protein/ml, 2 µM DPH). The mixture was incubated at 25°C for 30 min. After this period of incubation, pesticides were added from concentrated ethanolic solutions. The period of equilibration with pesticides varied from 1 to 2 h according to the concentration used. Control samples received an equal amount of tetrahydrofuran and ethanol. It should be stressed that concentrations of pesticide used are within its solubility range under the experimental conditions.

### Fluorescence Polarization Studies

Steady-state fluorescence polarization measurements were performed on a LS55 Luminescence spectrophotometer (PerkinElmer, www.perkinelmer.com), which is equipped with two photomultipliers to detect separately each polarized component of the fluorescent light. The temperature in the cuvettes was controlled with a thermostated circulating-water pump. Steady-state fluorescence anisotropy(r) and polarization (P) were obtained using the excitation and emission wavelength at 365 nm and at 426 nm, respectively, and excitation and emission slits with a nominal bandpass of 10 nm were used for all measurements. The mixture of DPH and PBS solution without added *P. xylostella*, mitochondrial membranes was used as a control. Polarization is inversely proportional to fluidity, relative fluidity was expressed as 1/P. The degree of fluorescence polarization (P) and fluorescence anisotropy (r) was calculated according to Shinitzky and Barenholz (1978) from the followed equation:

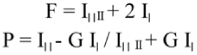

Where G is an instrumental correction factor, and I| and I‖ are, respectively, the intensities measured with the polarization plane parallel and perpendicular to that of the exciting beam.

### Statistical analysis

To estimate parameters of dose—mortality regression lines for each leaf-dip bioassay, data from the three replicates were pooled and analyzed with probit models using the POLO program (Russell et al. 1977). Two LC50 values were considered to be significantly different (*P* < 0.01) if their 95% fiducial limits did not overlap.

**Table 1.**
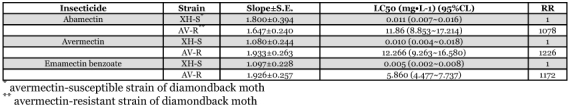
Cross-resistance of avermectin-resistant strain of *Plutella xylostella* to analogs of avermectins.

Other experimental data are expressed as mean values ± SE of experiments performed in triplicate. Statistical analysis was carried out using the one-way analysis of variance followed by the Newman-Keuls test. A value of P <0.05 was considered statistically significant.

## Results

### Cross-resistance

The strain of *P. xylostella* selected by exposure to avermectin, developed 1078-fold resistance to avermetin with a high level of cross-resistance (> 1000 times) to the analogs of avermectin, ivermectin and emamectin benzoate (Table 1).

### Effects of insecticides on fluidity of mitochondria membrane in XH-S *P. xylostella*,

DPH is quite a common and very well known probe for monitoring the fluidity of native membranes (Jone and Lee 1985; Antunes-Madeira et al. 1990a, 1990b, 1990c; Blasiak 1993). The absorption and emission spectra of DPH of mitochondrial membranes from *P. xylostella* larvae were measured. The absorption spectrum was centered at approximately 360 nm and the emission spectrum was centered at 430 nm. Both absorption and emission spectra are typical of those reported for DPH in other membrane systems (Shinitzky and Barenholz 1974, 1978).

The effect of different insecticides on the fluidity of mitochondrial membranes from larvae of the XH-S strain at 25°C was detected by fluorescence polarization (Table 2). The result showed that abamectin, emamectin benzoate, ivermectin, cypermethrin and fenvalerate can significantly increased the fluorescence polarization of DPH in gel phase, indicating that these insecticides may decrease membrane fluidity. No significant changes in the fluorescence polarization of DPH were found when mitochondrial membranea were incubated with fipronil and acephate, indicating that these insecticides could not change membrane fluidity when the concentration of these insecticides was 1×10^-4^ mol/L. However, endosulfan improved membrane fluidity, which was consistent with the previous studies (Martins *et al*. 1997).

**Table 2.**
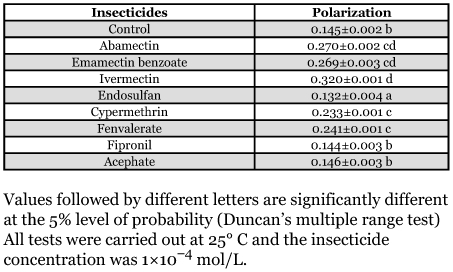
Effects of insecticides on fluidity of mitochondria membrane in the XH-S strain of *Plutella xylostella*.

**Figure 1.**
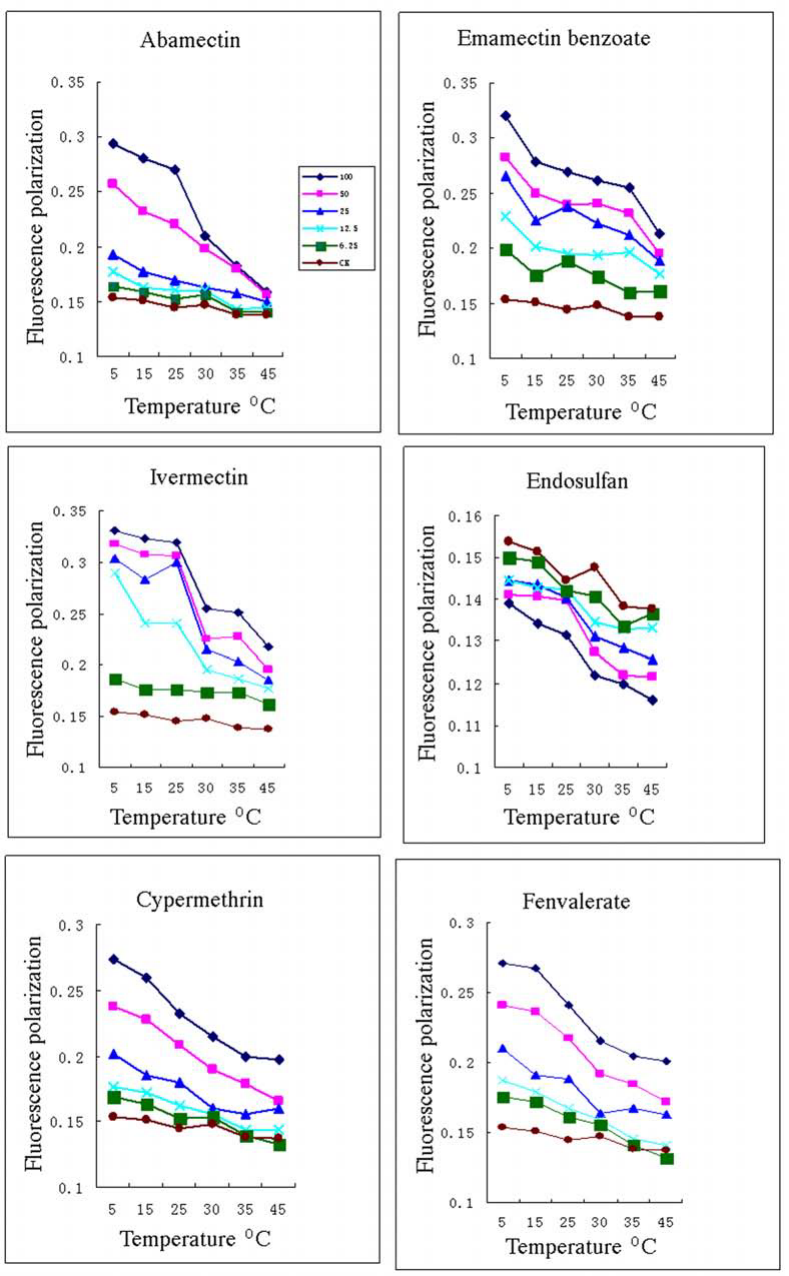
Thermotropic effects of various insecticides on membrane fluidity by fluorescence polarization in XH-S of *P. xylostella* larvae. The capital letters in the top of every figure represent different insecticides: A, abamectin; B, emamectin benzoate; C, ivermectin; D, endosulfan; E, cypermethrin; F, fenvalerate. Symbols indicate different concentrations to incubate with membrane.

The polarization value of DPH in mitochondrial membranes decreased with the increase of temperature regardless of the treatment with or without insecticides (Figure 1). The response to abamectin showed a significant dose-dependent increase of fluorescence polarization values in the gel phase that was consistent with the thermotropic transition phase (Figure 1), indicating that a condensing effect of the membrane is associated with the perturbation induced by abamectin. The same tendency was observed in induction of fluorescence polarization values with emamectin benzoate (Figure 1B), ivermectin (Figure 1C), cypermethrin (Figure 1E) and fenvalerate (Figure 1F), in mitochondrial membranes from *P. xylostella*. However, endosulfan induced a decrease in fluorescence polarization values of mitochondrial membranes (Figure 1D).

**Figure 2.**
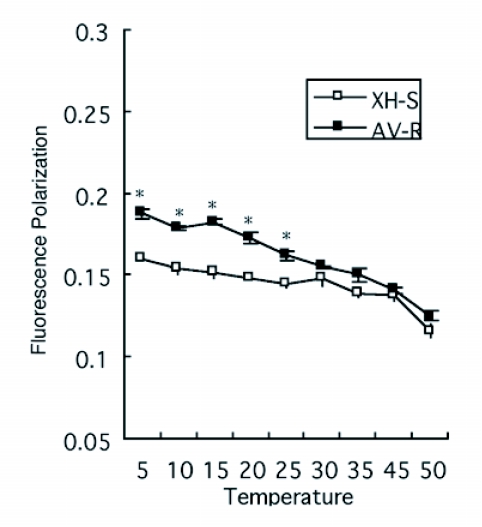
Thermotropic changes of fluorescence polarization in mitochondrial membranes from AV-R and XH-S *P. xylostella* larvae. *indicates significant differences between data from AV-R and XH-S (p<0.05).

**Table 3.**
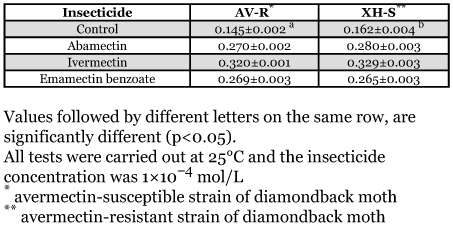
Comparison of fluorescence polarization between mitochondria membrane from AV-R and XH-S strains of *Plutella xylostella*.

### Comparison of membrane fluidity between XH-S and AV-R strain

Comparison of membrane fluidity between XH-S and AV-R strains at different temperature showed that the fluorescence polarization values of DPH in membranes were lower in XH-S strain than in AV-R strain at the tested temperatures (Figure 2). There was a significant difference in mitochondrial membrane fluidity between both XH-S and AV-R when temperature was less than 25°C and no difference was observed when temperature more than 25°C. When the temperature reached 30°C, the differences in polarization values of the mitochondrial membranes between XH-S and AV-R were no longer statistically significant. The thermotropic transition of mitochondrial membrane in *P. xylostella*, was about 30°C. But avermectin eliminated the difference of mitochondrial membrane polarization between XH-S and AV-R when the mitochondrial membranes were incubated with 1×10-4 mol/L of avermectin (Table 3).

**Figure 3.**
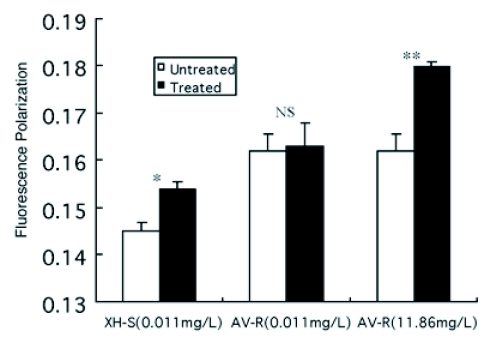
Fluorescence polarization of mitochondrial membranes from both untreated (white bars) and treated larvae of *P. xylostellas* (solid bars). The concentration 0.011 mg/L was the dose to kill 50% individuals of XH-S strain, but no lethal to AV-R strain. The concentration 11.86 mg/L was the dose to kill 50% individuals of AV-R strain. The *P. xylostella* larvae were treated by leaf-dipping method. The significance of the differences between the two groups was determined by the Student's t-test. The difference was considered significant at p<0.05, showed by *; for p<0.01, showed by **; NS, not significant.

The effects of abamectin on the *P. xylostella in vivo* were also studied after larvae were fed on cabbage leaf treated with abamectin for 6 hours. Figure 3 shows the fluorescence polarization when DPH was added to membranes from both untreated and abamectin-treated *P. xylostella*. After larvae fed on cabbage leaf treated with 0.011 mg/L abamectin, fluorescence polarization was higher when DPH was added to membranes from abamectin-treated XH-S strain when compared with untreated XH-S strain, but no significant difference was found between abamectin-treated and untreated AV-R strains. However, when the AV-R strain was fed on cabbage leaf treated with 11.86 mg/L abamectin, fluorescence polarization of membranes from treated larvae of *P. xylostella* was much higher than untreated *P. xylostella* (Figure 3). These results showed that abamectin induced mitochondrial membrane fluidity of *P. Xylostella in vivo.*

## Discussion

The effect of pesticides on biomembrane has been studied in rats, human cells and microorganisms. Rosita et al. (2002) found that the pyrethroid, cypermerthrin, induced a significant decrease in erythrocyte membrane fluidity and a similar result was also found by Cinzia et al.(2003). However, endosulfan, an organochlorine insecticide, may improve membrane fluidity of *Bacillus stearothermophilus* (Martins et al. 2003). In accordance with these previous studies, the pyrethroids decreased the fluidity of mitochondrial membranes of *P. xylostella*, while endosulfan increased fluidity in our studies. The effects of avermectins and fipronil on membrane fluidity have not been found before our studies and these pesticides also decreased the membrane fluidity. These effects on membrane are certainly related to the chemical structure of the pesticides. Compared with pyrethroids, the avermectins are more hydrophobic, which may relate to the result that avermectins resulted in an increase in polarization value.

Membrane fluidity may vary with the concentration of pesticides (Antunes-Madeira et al. 1990a; Rosita et al. 2002). This concentration-dependent effect reflects a decrease in the molecular packing promoted by the perturbation of the membrane order induced by the insecticides (Suwalsky et al. 1997). These insecticides may cause the structural alteration in mitochondrial membranes and these changes may relate to the ion currents caused by different insecticides. The region involved may be the bilayer hydrophobic region and to a lesser extent the more superficial or polar regions, an observation in agreement with previous reports (Buff and Berndt 1989; Suwalsky et al. 1997). The most obvious effects produced by nearly all the pesticides studied was a concentration-dependent effect, which reflects the similarity in mitochondrial membranes between insects and other organisms.

Resistance is a microevolutionary process of genetic adaptation in response to insecticides (Whalon and McGaughey 1998) that can result in the failure of a plant protection. The mitochondrial membrane may be a major target of insecticides, and may, therefore, play a critical role in the development of resistance. Tabarean et al. (1998) found that the deltamethrin and tetramethrin share a binding site on the sodium channel, and that the slow onset and offset of deltamethrin action are controlled by the rates at which deltamethrin moves and unbinds from the membrane lipid phase rather than by the rate of deltamethrin binding to the sodium channel site based on tetrodotoxin-sensitive and tetrodotoxin-resistant sodium channels, which showed the importance of membrane structure in deltamethrin resistance. In our studies, mitochondrial membrane fluidity was significantly lower in the AV-R strain than in XH-S strain, but no difference was found when these strains were exposed to avermectins. This indicates that the AV-R strain showed a resistance to membrane disrobing by abamectin. The mitochondria membrane structure in AV-R may be altered, which may relate to its resistance to avermectin. There are several mechanisms to explain the effects of avermectins on membrane fluidity. First, a direct action of the avermectins on the lipid bilayer could occur that modifies lipid-lipid interactions and causes leaky vesicles (Buff et al. 1981). Second, an association of the insecticide with lipids surrounding transmembrane domains may affect protein orientation and the properties of these particular membrane domains. Third, an accumulation of avermectins in the hydrophobic domains of integral membrane proteins, may altered protein folding and/or protein-protein interactions (Jones et al. 1985; Michelangeli et al. 1990). The differential actions of tested insecticides on the GABA receptor-ionophore complex support the notion that several mechanisms might be involved in the interaction of different insecticides with mitochondrial membranes.

## Abbreviations

DPH -: 1,6-Diphenyl -1, 3, 5-hexatriene
XH-S -: avermectin-susceptible strain of *P. xylostella*
AV-R -: avermectin-resistant strain of *P. xylostella*
